# Semantic-Enhanced Multi-Dimensional Markov Chains on Semantic Trajectories for Predicting Future Locations †

**DOI:** 10.3390/s18103582

**Published:** 2018-10-22

**Authors:** Antonios Karatzoglou, Dominik Köhler, Michael Beigl

**Affiliations:** 1 Karlsruhe Institute of Technology, 76131 Karlsruhe, Germany; dominik.koehler@student.kit.edu (D.K.); michael.beigl@kit.edu (M.B.); 2 Robert Bosch, Corporate Sector Research and Advance Engineering, 70049 Stuttgart, Germany

**Keywords:** semantic trajectories, semantic location prediction, semantic similarity, multi-dimensional markov chains, context awareness

## Abstract

In this work, we investigate the performance of Markov Chains with respect to modelling semantic trajectories and predicting future locations. In the first part, we examine whether and to what degree the semantic level of semantic trajectories affects the predictive performance of a spatial Markov model. It can be shown that the choice of the semantic level when describing trajectories has a significant impact on the accuracy of the models. High-level descriptions lead to better results than low-level ones. The second part introduces a multi-dimensional Markov Chain construct that considers, besides locations, additional context information, such as time, day and the users’ activity. While the respective approach is able to outperform our baseline, we could also identify some limitations. These are mainly attributed to its sensitivity towards small-sized training datasets. We attempt to overcome this issue, among others, by adding a semantic similarity analysis component to our model that takes the varying role of locations due each time to the respective purpose of visiting the particular location explicitly into consideration. To capture the aforementioned dynamics, we define an entity, which we refer to as Purpose-of-Visit-Dependent Frame (PoVDF). In the third part of this work, we describe in detail the PoVDF-based approach and we evaluate it against the multi-dimensional Markov Chain model as well as with a semantic trajectory mining and prefix tree based model. Our evaluation shows that the PoVDF-based approach outperforms its competition and lays a solid foundation for further investigation.

## 1. Introduction

According to recent statistics published by eMarketer [1], 242 million people in the USA are expected to use location-based services (LBS) in 2018. This number corresponds to almost double the number of LBS users five years ago, in 2013 (123 millions). While typical context-aware applications, such as LBS, are focusing on reacting to users’ location-dependent needs, current approaches strive to behave in a forward-looking manner to raise the quality of their service. This *Proactivity* is a key feature, which leads to a great number of benefits including higher efficiency and an improved human–machine interaction. Efficient asset and resource management, such as the hand-off management in mobile communications networks, and timely offerings of services and solutions, which have been tailored to the user, such as the ones provided by digital personal intelligent assistants, both rely to a large extent on the feature of proactivity. Consequently, the significance and value of location prediction with respect to the domain of location-based services and of context-aware systems in general is self-explanatory.

Location information reveals to us humans more than just the *whereabouts*. It gives indirectly insight about the *what* and the *when*. While the *what* refers to the activity and the overall purpose of visiting a certain location, the *when* provides the temporal information, such as time, day and date; frequency; and periodicity. For instance, the location *night club* is put usually in context with some overall, semantically high-level purposes, such as *socializing*, *having fun*, *chilling*, *relaxing*, and a set of elementary, lower-level activities, such as *drinking*, *dancing*, and *meeting friends and/or new people*. Moreover, a human would additionally associate some corresponding temporal information with it, such as *night*, *weekend*, *once a week* and maybe *all night long*. For humans to be capable of interpreting locations at such a high-level and associating them with all this additional information, we rely both on a broad framework of semantics hidden behind them, as well as on a large portion of world and common sense knowledge. At the same time, *each human takes his/her own personal experience and knowledge into consideration*. This agrees also with the general definition of *knowledge*, which is defined in [2] as:

*Facts, information and skills acquired through experience or education; the theoretical or practical understanding of a subject.*

A generic semantic framework, together with a common sense knowledge base, provides a mutual basis among different people for interpreting things similarly. In comparison, *personal experience can rather lead to different interpretations among people*. Let us clarify this in the location scenario by going back to the example mentioned before. In the particular example, the location *night club* was interpreted from the perspective of a guest, which is the most common one. However, for the barkeeper, a club is a *working location*. It is bound now to a completely different high-level aim and set of elementary actions, such *working/earning money*, and *making drinks*, *serving drinks*, *talking to the customers* and *getting paid*, respectively. A similar ambiguous effect would arise in the case of a restaurant between a guest and the cook or the waiter working there.

Let us now consider another example. The location *hotel* is for a tourist obviously closely linked to a stay location over the holidays, while for the receptionist it is a place of work with highly fixed attendance times. People who are visiting a conference or are having a business lunch there would also experience the hotel from a similar perspective during that time, since their visit is of professional nature. On the other hand, if the same people would enjoy a drink at the bar of the same hotel after their business meeting is over, they would associate the hotel rather more with a night life location, similar to a *bar* or *club*. This example highlights another important issue, namely that people tend to perceive, interpret and associate locations to each other *dynamically, depending on the situation, in which they find themselves*. Nathan et al. supported this theory with regard to human movement analysis by interpreting movement between locations as the outcome of the synergy of four components [3]: the internal state of the individual, their motion capacity, their navigation capacity and potential external factors, whereby the internal state addresses the situation in which a person finds himself. This kind of location-specific “semantic ambiguity” can mostly be found in multipurpose locations, a fact that was confirmed in the study presented in Section 5.1.2 as well. That is, locations, which offer a variety of reasons to visit them, such as the mall (*shopping*, *getting a haircut*, *meeting a friend*, etc.), the hotel (as described in the aforementioned example), the park (*picnic*, *jogging*, *sitting on the bench reading a book*, etc.), our home (*working*, *relaxing*, *celebrating*, *eating*, etc. Summarizing the above leads us to the following two aspects as already stated in [4]:

1. The same location potentially has different meanings to different people.
2. A location may even have many different meanings to the same person depending on the situation.

Location prediction algorithms that utilize semantics and rely on so-called *semantic trajectories* (see Section 3.1) go beyond plain numerical data, such as GPS coordinates and Cell-ID sequences. The use of semantics gives them a number of advantages. The most significant one is the fact that semantic trajectories carry more knowledge with them and are capable of capturing the essence of human movement patterns. This can be particularly helpful for a location predictor in places that have not been visited before by the users and for which there are therefore no GPS recordings available on which the predictor could be trained. Beyond that, semantically enhanced location prediction systems gain transparency through the use of semantics. On the one hand, this leads to a better human–machine relationship. On the other hand, it assists indirectly the compliance with the data protection regulations. Finally, and not unimportant in the age of the Internet of Things (IoT), semantics and particularly knowledge sharing tools such as the associated knowledge graphs and ontologies provide a robust basis for machine-to-machine (M2M) communication. This is vital for creating independent, fully autonomous and intelligent environments.

However, up to this point and to the best of our knowledge, none of the semantic trajectory based approaches have taken the *varying role and human perception of locations* into account. Instead, they constrain themselves to static semantic location types and inflexible associations between locations and users as described in the related work section below (Section 2). In this work, we describe a semantic trajectory based location prediction approach that considers explicitly the dynamic, purpose-of-visit-driven varying role of locations to achieve a higher performance. Our approach relies on the hypothesis that *locations resemble one another from the point of view of the user in relation to the purpose of visit* and that *similar locations come also with similar transitions from one location to another as well*. In tangible terms, we hypothesize that a person who always visits a *take away restaurant* after visiting the *gym*, will also visit some *food location* too after having a similar fitness activity such as climbing in a *boulder hall* or jogging at a *park*. While the *gym* and the *boulder hall* are associated with each other and belong to the same high-level location type, *fitness location*, this holds only exceptionally for the *park* case, as it *shares the same purpose of visit*. The core idea of the approach presented here lies in a dynamic and context-aware clustering of semantic locations. For this purpose, we combine two different modelling techniques, a *data-driven* one and a *knowledge-driven* one, by using the *semantic similarity analysis* as a fusing component.

This work relies on our previous investigations [5] and extends them as follows. First, it provides a more thorough analytical view upon the related work and the fundamental components of our approach (Section 2, Section 3, Section 5 and Section 6). Furthermore, in Section 4, in contrast to our previous work, we investigate the impact of using different semantic representation levels on the predictive performance of our model. For this purpose, we compare two different representation levels by mapping the available locations in our training and evaluation dataset correspondingly (e.g., *burger joint* to *restaurant*). In Section 5, we extend the spatial Markov model by taking both time and activity additionally into account and we compare it with our initial vanilla spatial model. Finally, in Section 6, we implement and evaluate, besides the multi-user model in [5], the respective single-user variants of our aforementioned semantic-similarity enhanced PoVDF-based approach as well, by comparing them among other against the semantic trajectory mining and prefix tree based approach of Ying et al. [6]. Moreover, we explore how individual features (e.g., time, day, activity, etc.) and their combinations affect the performance of the aforementioned models. We show that our final approach is able to converge more towards human movement patterns and can therefore lead to a higher predictive performance compared to other semantic trajectory based approaches. To evaluate our models in a real-world scenario, we designed and carried out three user studies, where we collected semantically annotated tracking data from a single user over a period of 12 weeks as well as from 21 users over a period of eight weeks and from 10 mobile users over a period of five weeks, respectively. These are described in Section 4.1 and Section 5.1.1. Finally, in Section 8, we summarize our findings and discuss some thoughts about potential future work.

## 2. Related Work

Probabilistic models represent a very popular approach when it comes to modelling trajectories and provide a solid basis for accurate predictions of mobile users’ future moves. Markov models are especially common among the respective literature due to their computational efficiency. Some use Markov models use solely spatial information and some include additional context information, such as time or transportation mode, into their models as well. The first part of this section provides a brief insight into some of the most related work that utilize, among others, various types of Markov models, to predict the next location of a user. The second part refers to some works that, although they do not come from the same field, their semantic similarity and relatedness based approaches served as a basis for our own model.

Ashbrook et al. and Gambs et al. investigated in their work the performance of traditional Markov models with regard to location prediction accuracy [7,8]. Their results show that the order of the model may have a significant impact on the accuracy. High order models seem to perform better than lower order ones. However, choosing a too high order affects the model adversely. Asahara et al. demonstrate in [9] that a mixed Markov model (MMM), which considers users with similar movement behaviour to fall within respective groups, performs better than a simple Markov model (MM) and a Hidden Markov model (HMM). Ye et al. adapted and applied in their work Altman’s mixed Hidden Markov model (MHMM) [10] on semantic locations coming from a location based social network (LBSN) [11]. Their mixed model incorporates both spatial and temporal information by (statistically) mapping location categories to a number of predefined time periods (e.g., *bar* with *night*). In addition, they clustered the users based on their movement and activity patterns. Finally, a separate model is trained for each cluster of users. It is shown that time as an additional context information as well as the clustering of similar users helps to raise the prediction accuracy. Wesley et al. [12] considered temporal as well as spatial information. They proposed a triple Hidden Markov model (HMM), that is, a set of 3 HMMs, with each modelling spatial information coming only from one of the following temporal periods respectively: weekdays from 07:00 to 19:00, weekdays from 19:00 to 07:00, and weekends. Similar to Ye et al., they showed that this kind of *temporal attention* can lead to an improved prediction accuracy.

Ying et al. [6] went in another direction and used their own Geographic Semantic Information Database (GSID) to enrich semantically their recorded GPS- or Cell-ID trajectories. GSID is a customized POI (Point of Interest) database that stores semantic information of landmarks, geographic scopes and associated location types. From the resulting semantic trajectories, Ying et al. mined the most significant patterns, which in turn they converted into Semantic Pattern Trees to finally provide the basis for the next place prediction. In [13], they extended their approach by taking temporal information into account as well. The work of Karatzoglou et al. focuses on modelling semantic trajectories as well. In [14], they proposed a personalized matrix factorization based model to overcome the sparseness of the available training and evaluation datasets. Their recent work [15,16,17] explores the use of deep and shallow artificial neural network architectures, from simple Feed Forward neural networks (FFNN), to Convolutional neural networks (CNN) as well as recurrent types, such as Long Short-Term Memory models. Furthermore, they investigated the use of embedding layers and auto-encoder architectures in the example of Sequence to Sequence Learning. Yao et al. [18] proposed and evaluated a recurrent neural network model with embeddings as well with equally promising results. Samaan et al. used spatial conceptual maps to describe buildings and road network elements semantically [19]. In addition, a user context knowledge base formulated in XML, which contains the users’ preferences, schedule, tasks and goals, further supports the location prediction. Their mobility prediction algorithm is probabilistic and relies on the Dempster–Shafer Theory [20]). In [21,22], they illustrated the same algorithm, only that now the locations are represented by Cell-IDs assigned by the corresponding cell towers. Ridhawi et al. applied a similar algorithm for tracking and predicting users indoors to support location-aware services in [23,24]. Their algorithm also uses the Dempster–Shafer Theory for returning the final future location estimation. However, in contrast to Samaan et al.’s algorithm, the knowledge is structured and stored by means of OWL-based ontologies. These ontologies contain the profiles of the users, their location history and some activities.

Long et al. [25] proposed a location clustering algorithm, which uses the Latent Dirichlet Allocation method, a probabilistic model used normally to cluster documents based on the topics contained in them. They defined so-called *geographic topics* in an unsupervised manner from the check-ins at the corresponding venues of Foursquare (https://foursquare.com/) users based on their popularity. These topics replace static location categories such as the ones provided by Foursquare. Additionally, they investigated how location clusters behave depending on whether it is a weekday or weekend. In [26], Krishnamurthy et al. exploited Twitter (https://twitter.com) tweets to predict the location of its users. They introduced the concept of *localness* to express how “close” certain terms appearing in a tweet, so called *local entities*, are to particular cities. To this effect, they investigated several different measures, including two semantic relatedness measures, the Jaccard [27] and the Tversky [28] Indices. After determining the *localness* scores for each city of the corresponding local entities in the tweets of a user, it is possible to estimate the location of the user. Finally, Wannous et al. and Malki et al. proposed a multi-ontology based approach in combination with a set of rules created by a group of experts to model and reason about movement and activity patterns of marine mammals [29,30,31,32,33]. Their set of rules is subdivided into spatial, domain-specific and temporal rules respective each time to the applied ontologies.

In [34], Mabroukeh et al. utilized first semantic information to assist the sequential web usage pattern mining process. Then, they used semantic relatedness for determining the transition probabilities of a Markov Model that predicts the next page visited by the user. Zhao et al. proposed a time-dependent semantic similarity measure of web search queries by considering the temporal factor when mining click-through data to express the dynamic nature of queries over the time [35]. In addition, they placed their trust in a probabilistic similarity measure that reflects the web queries’ frequency distribution.

All aforementioned approaches constrain themselves to static location categories and types without considering the dynamic and purpose-of-visit-dependent varying role of locations to the users. This *lack of flexibility* is carried over to their methods of representing associations between locations and users through static and unalterable axioms or rules. Solely, Long et al.’s work investigates a dynamic approach but it is based only on popularity and not on the semantics behind the locations. In this work, we introduce a location prediction model that also takes the aforementioned dynamics into account. We show that, by doing so, we are able to improve the model’s precision and accuracy.

## 3. Background Theory

This section covers the theory behind the basic concepts presented in this work.

### 3.1. Semantic Trajectories

The term *trajectory* refers to spatiotemporal sequences which define the movement of objects or persons under a given reference system (*frame*) during a certain time interval. GPS or Cell ID sequences are such sequences. Equation (1) shows a common GPS trajectory, where each point in the trajectory is being described by a quadruple containing its latitude, its longitude, the altitude and the corresponding time:

(1) $$traj=\left(lat_{1},long_{1},alt_{1},t_{1}\right),\left(lat_{2},long_{2},alt_{2},t_{2}\right),\ldots$$

However, to understand the logic behind such movement patterns and the behaviour of the travelling objects themselves, and to be eventually capable of predicting their next steps and act upon them accordingly, we need to go beyond plain position data and acquire more knowledge about the respective trajectories. Spaccapietra et al highlighted first the importance of utilizing *semantics* when analyzing trajectories [36] and introduced a *conceptual view* over them. In their work, they point out the varying underlying purpose and semantical meaning of trajectories. At the same time, basic semantic elements such as *stops*, *moves*, *begin* and *end* are being defined. Each item in a semantic trajectory represents a *significant* location [37], a location at which a user is staying for more than a certain amount of time to carry out a certain activity, whereas the type of the activity affects the size of the respective time window. The notion of these *semantic* locations and their role in human mobility patterns conforms in a way with Hägerstrand’s concept of a *space-time prism* defined by a set of so-called *space-time anchors* [38] that describes the trade-off between time and space depending on the respective activity. However, Hägerstrand’s approach is physical and views human mobility from a constraint satisfaction problem solving view. That is, it handles additional context information, such as activity, speed and the users’ mobility capability, explicitly as constraints to derive the potential paths of a human user. This is done in the case of semantic trajectories and in our work in particular rather implicitly, as shown below. In [39], Yan et al defined a *semantic trajectory* as

“A structured trajectory where the spatial data (the coordinates) are replaced by geo-annotations and further semantic annotations”.

resulting to a sequence of *semantic episodes*:

(2) $$traj_{sem}=se_{1},se_{2},se_{3},\ldots$$

**Figure 1:**
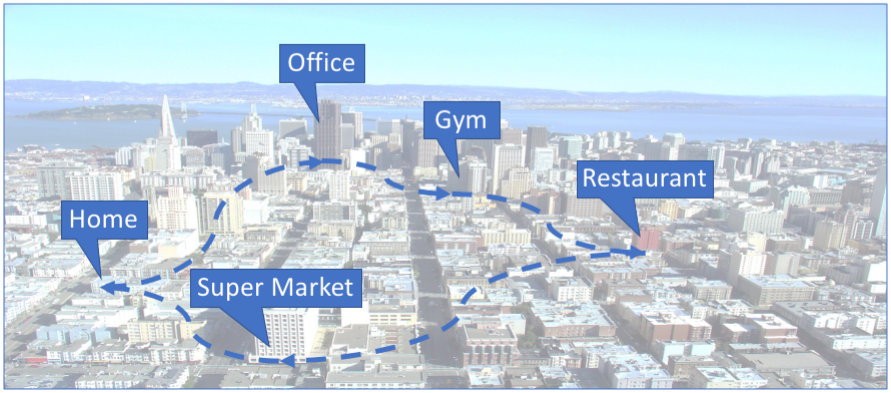
Example of a one-day long Semantic Trajectory. (Image based on: https://commons.wikimedia.org/wiki/File:San_Francisco_downtown.jpg).

Figure 1 illustrates this particular semantic trajectory.

The prediction models introduced in this work rely on exactly this type of semantic trajectories. Moreover, we explore the impact of modelling trajectories at different semantic representation levels on their performance (see Section 4).

### 3.2. Markov Chain Model

A *stochastic process* describes an ordered set of one or more random variables and is commonly used to define dynamic processes that change randomly over time. A *Markov Chain* model (or simply *Markov Chain* or *Markov model*) defines a memoryless stochastic process; a stochastic process, which additionally satisfies the Markov property. According to the Markov property, predictions about a future state based on a short history lead to similar results to those based on a longer or the whole history. Markov Chains can be classified by their *order*, which determines how far back history is taken into account. A 1. order Markov Chain is determined by the following conditional (Markov) property [40]:

(3) $$p\left(z^{\left(m+1\right)}|z^{\left(1\right)},z^{\left(2\right)},\ldots ,z^{\left(m\right)}\right)=p\left(z^{\left(m+1\right)}|z^{\left(m\right)}\right),$$

where *z^(1)^*, *z^(2)^*, … is a series of successive random state variables (or just *states*) and *p* represents the *state transition probability* value. Thus, the prediction relies in this case solely on the current state and is independent from the former ones. A 2. order Markov model would analogously consider both the current and the previous state, and so on. Higher order Markov Chains tend therefore to cluster the considered previous states together. In this work, the states correspond to semantic locations $L=\{l_{1},l_{2},\ldots \}$ and the Markov Chain model is used to model the respective semantic trajectories $T_{sem}$ and subsequently to predict the future movement behaviour of the users U = $\left\{u_{1},u_{2},\ldots \right\}$.

**Figure 2:**
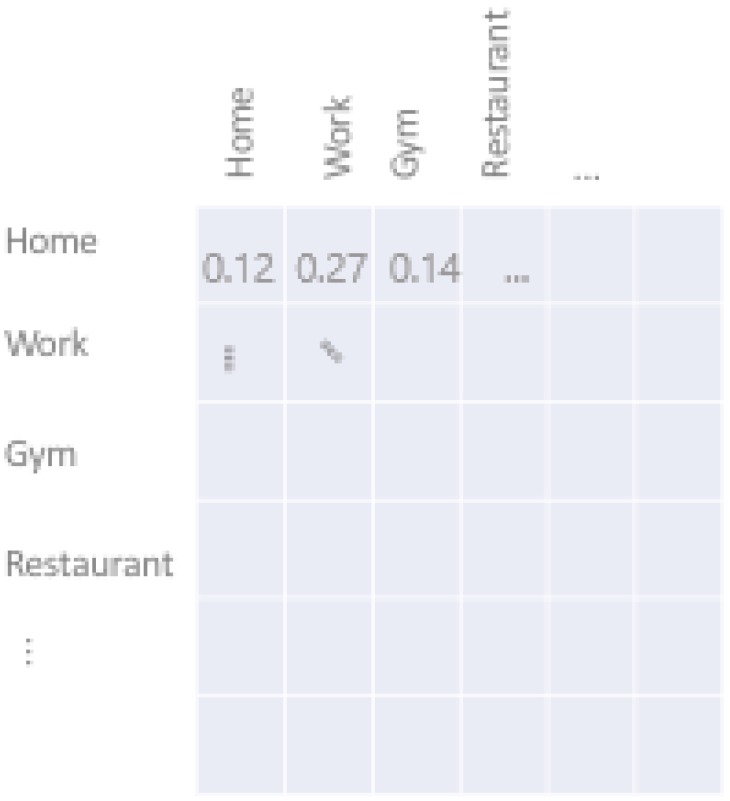
Semantic location transition probability matrix.

In general, a Markov model can be described through its *state transition probability matrix*, or simply *transition matrix A*. Each element of the transition matrix contains the probability for changing from a certain state to another. In our case, a state transition refers to moving from a certain semantic location to another location. Figure 2 illustrates an example of such a semantic location transition matrix. Each row and column represents a single semantic location.

According to this matrix, there is a 27% chance for a user being at home to visit the gym next, while there is only a 12% chance to stay home. The transition matrix can refer either to a single user or to a set of users.

## 4. Applying a Spatial Markov Chain Model on Semantic Trajectories at Different Semantic Levels

As shown in Section 3.1, semantic trajectories can be described in various ways depending on the semantic level used at each time. Furthermore, the choice of the semantic level determines the modelling granularity. This section explores the impact of the semantic level on the modelling performance of a 1. order spatial Markov Chain model. In particular, in this section, we compare and evaluate two different semantic levels, a low- and a higher-level one. To define these two levels, we oriented ourselves on the Foursquare venue taxonomy (https://developer.foursquare.com/docs/resources/categories). The Foursquare venue taxonomy defines a five-level hierarchical construct of location categories with the following 10 major categories being at the top level:

- Arts (Culture and Entertainment)
- College and University
- Events
- Food
- Nightlife
- Outdoors (Nature and Freetime)
- Professional and Other Locations
- Residence
- Shop and Services
- Travel and Transport

Each of the top-level categories comprises a set of subcategories such as the one seen below:

- Food location
  - Greek restaurant
    ∗ Ouzeri
    ∗ Tavern
      ⋅ Fish tavern
      ⋅ …

In this section, we compare the top semantic level ($Level^{\left(1\right)}$) with the third level ($Level^{\left(3\right)}$), e.g., *Food location* vs. *Tavern* or *Nightlife* vs. *Bar*. The choice fell on these particular levels in part due to the range and the distribution of the annotated location types found in our training and evaluation datasets, which are described in the evaluation Section 4.1 below.

### 4.1. Evaluation

We used two different real-world datasets in our evaluation, a 12-week long single-user dataset and an 8-week long dataset with 21 participants. The first dataset is diary-based and contains the annotated daily trips of a single user during a period of three months. The second dataset is based on an experimental field study, in which 21 mobile users were tracked for a period of two months and is described in detail in [41]. Our datasets include all 10 high $Level^{\left(1\right)}$ location categories and a total of 70 different low $Level^{\left(3\right)}$ location types. To evaluate the Markov model in terms of prediction with regard to the semantic level, we applied a 10-fold cross validation for each semantic level. Table 1 provides the average and the maximum recorded accuracy of a 1. order Markov Chain model after 10 shuffled runs for the respective two semantic levels for the single-user dataset case. It can be seen clearly that the Markov model performs better with high-level trajectories than with lower ones. It achieves an average accuracy of 75% and a maximum accuracy of 100% compared to 61.1% and 94.2% the low-level model, respectively.

The results for the multi-user dataset are set out in Table 2. Apart from the accuracy, Table 2 includes the weighted F-Score as well. The results follow a similar trend as before, with the high-level model ($Level^{\left(1\right)}$) outperforming the low-level one ($Level^{\left(3\right)}$) in terms of both average and maximum accuracy. The same trend can also be identified with respect to F-Score.

The outcomes in both cases can be attributed to the fact that people tend to move in space based on rules and patterns of higher semantical order. For instance, a person might regularly visit a *food location* after going to *gym* regardless of whether it is a *pizza house*, a *burger joint* or some *snack bar*. At the same time, a high-level scenario clusters low-level location types together and brings therefore less classes for the Markov model to predict. For this reason, a better performance in favour of the high-level trajectory model can be additionally expected.

## 5. A Multi-Dimensional Markov Chain Model for Modelling Semantic Trajectories

In this section, we propose a multi-dimensional Markov Chain model for modelling semantic trajectories and predicting future locations that takes both time and the user’s activity into consideration. For this purpose, we extend in a certain way the work of Wesley et al. [12] and build an ensemble of Markov Chains with each of them corresponding to a specific feature configuration with regard to type and value, which we refer to as *dimension*. In tangible terms, we define a multi-dimensional construct that comprises a set of transition probability matrices, one for each tuple combination *(time of day, day of week, activity)*. We regard time of day in 24 hourly time slots. Additionally, we cluster all annotated activities found in our training and evaluation dataset (see Section 5.1.1) into the following 12 high-level activities based on their content and frequency of occurrence:

- Working
- Training
- Trading
- Creating
- Shopping
- Socializing
- Celebrating
- Eating
- Travelling
- Getting ready
- Relaxing
- Visiting a doctor

**Figure 3:**
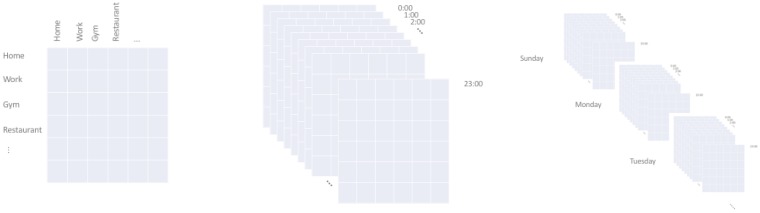
From left to right: Location transition probability matrix, time-of-day-specific transition probability matrices, and day-of-week-specific sets of transition probability matrices.

Figure 3 illustrates on the left an example of a location transition matrix as we had in Figure 2, while the middle and the right part elucidate vividly the multi-dimensionality of our model regarding time of day and day of week (24×7=168 matrices).

If we also consider the 12 activities, we come up to a total of 24×7×12=2016 transition matrices. This would require a great amount of data to cover all the possible combinations and get the elements of all transition matrices filled. Even if we had many data, some combinations might still not exist (e.g., (*03:00, Tuesday, doing fitness*)). To increase our chances of obtaining reasonably occupied transition matrices, we cluster the time into the following three time blocks:

- Morning: 05:00–11:59
- Midday: 12:00–16:59
- Evening-Night: 17:00–04:59

This results in a final total of 3×7×12=252 transition matrices, that is, 252 Markov Chains. Each of them is trained separately and covers a specific context scenario. During the prediction phase, our model picks out the appropriate Markov Chain depending on the current context (time, day, and activity).

**Figure 4:**
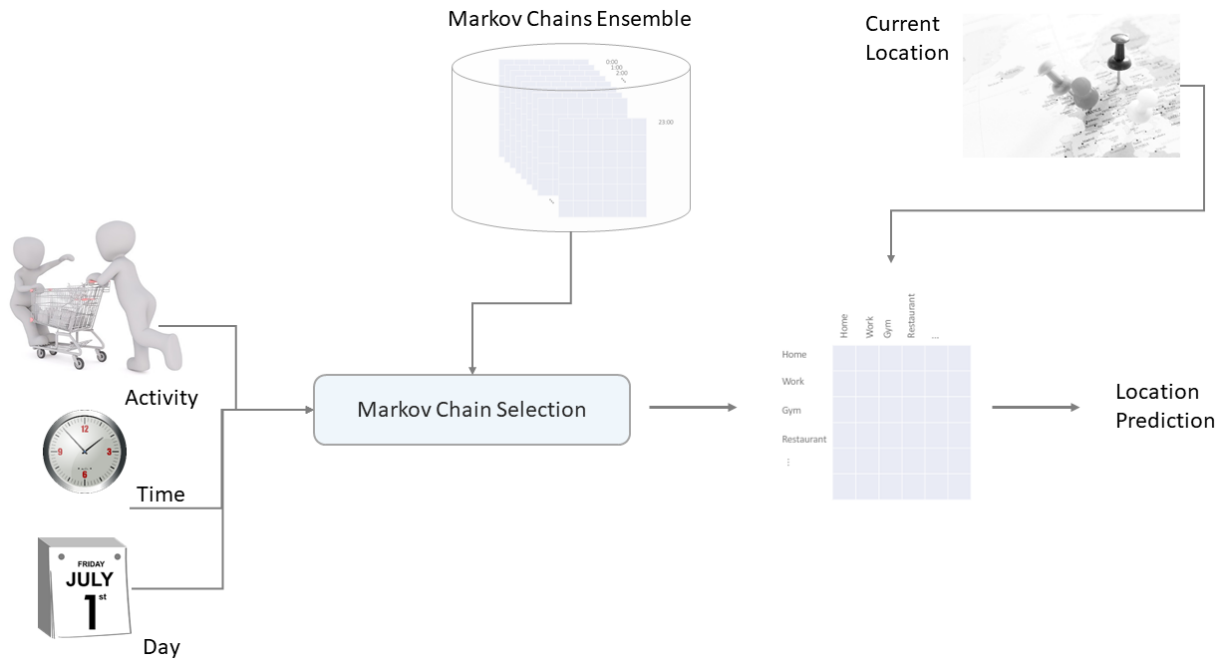
Markov Chain selection process and location prediction.

Figure 4 displays the Markov Chain selection process based on the additional context information (time, day and activity) and the subsequent location prediction based on the current location of the user.

### 5.1. Evaluation

This section covers the outcome of our multi-dimensional Markov Chain approach. While the first part refers to our training and evaluation dataset and how this was collected, the second part discusses in detail our evaluation results.

#### 5.1.1. User Study

**Figure 5:**
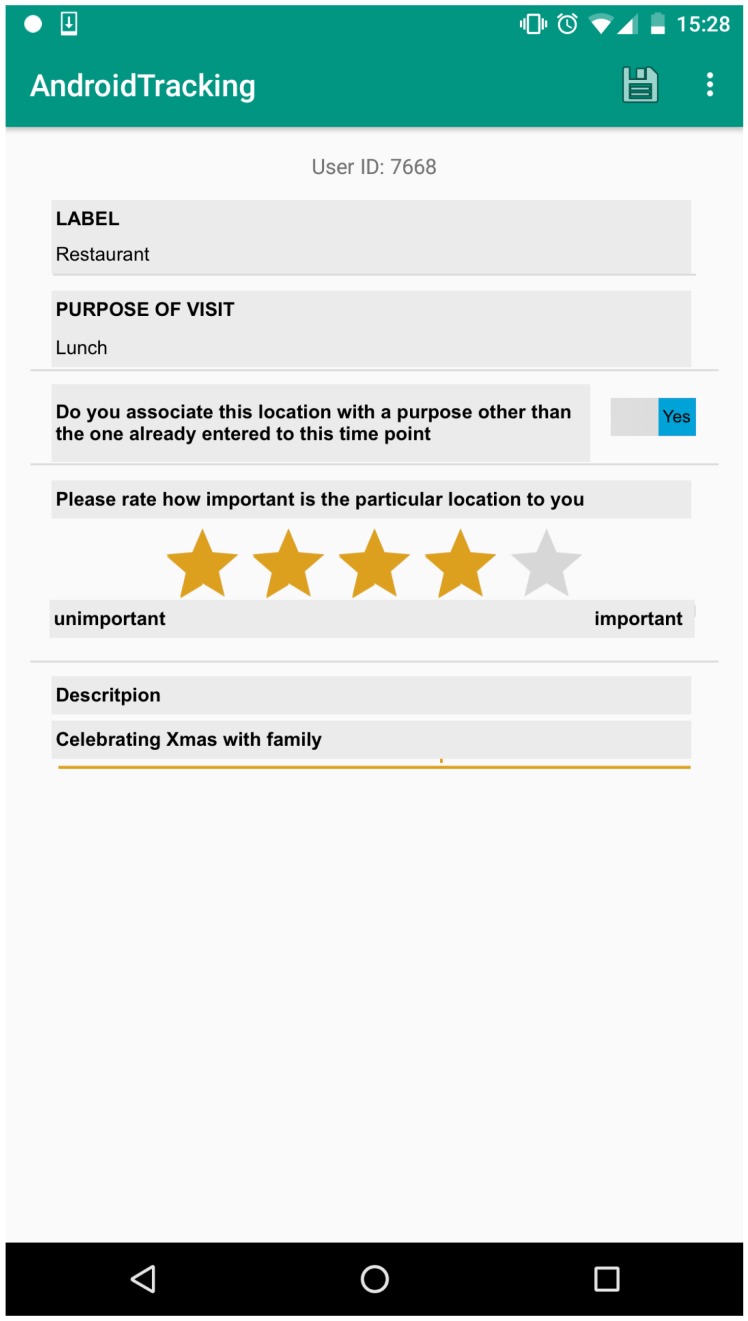
Screenshot of our Android tracking and annotation app.

To evaluate our multi-dimensional Markov Chain ensemble on a real-world dataset, we carried out a user study, in which we tracked the movement of 10 mobile users for a period of five weeks. Our participants aged from 18 to 28 years old and were mostly students. A tracking and annotation Android app was designed and implemented in order to collect the users’ data. For this purpose, we used the AWARE instrumentation and context logging framework (http://www.awareframework.com), which brings many benefits, such as an activity-based energy efficient and phone battery saving tracking algorithm. This is particularly important, since a power hungry app could affect adversely our study in two ways. On the one hand, it could lead to users closing the app and therefore missing valuable data. On the other hand, it might even influence their movement patterns, if the users had to plan more time somewhere in between for charging their smartphone. Thus, building an energy efficient app was a basic prerequisite for us. Figure 5 shows a screenshot of our Android app.

The users were able to pause, or close completely the app at any time. Each user was assigned an anonymous and random generated identification number (ID) to preserve the users’ privacy. The data were collected and stored first locally, on the mobile phone of the user, and sent encrypted to our server at the end of the study.

During the study we asked the users to:

- Label their location
- Enter the purpose of visiting the certain location (high-level activity)
- Define whether the particular location had been visited for another purpose up to that point
- Rate how important the particular location is to them, and
- Provide additional descriptive information, e.g., “sitting in a coffee shop with a friend after work”

Furthermore, three Amazon vouchers were raffled among the participants to increase their motivation.

#### 5.1.2. Results

**Figure 6:**
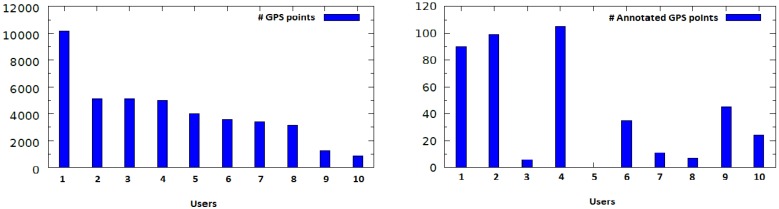
(**Left**) Number of tracked GPS points per user; and (**Right**) Number of annotated tracked GPS points per user.

Our user study provided us with an average of approximately 4000 GPS entries per user. However, the number of annotations varies strongly among the users. Figure 6 contains the distribution of the number of the tracked GPS points, as well as the number of the annotated tracked GPS points (locations) among the users.

**Figure 7:**
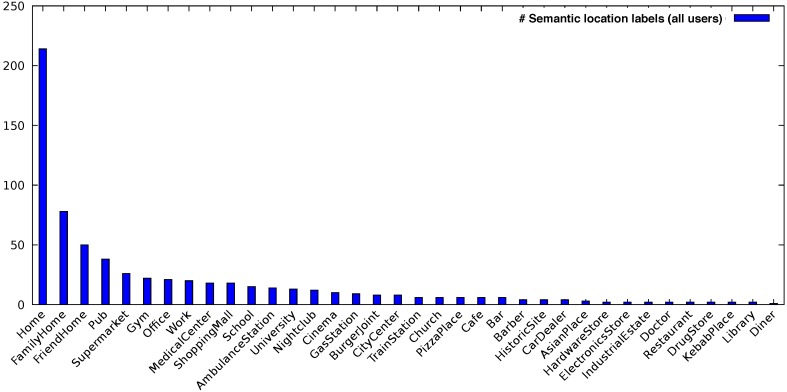
Semantic label distribution of locations.

It can be seen that Users 3, 5, 7 and 8 provided the least annotations. For this reason, we filtered these users out and our evaluation focused on the remaining six users. After analyzing their data, we over all had 431 different purpose-of-visit entries with respect to the following nine high-level location types: *Residence/Home*, *Travel and Transportation*, *Nightlife*, *Shopping*, *Services*, *Food*, *Freetime*, *Education/University* and *Work*. As before, we oriented ourselves on the Foursquare location taxonomy described in Section 4 to categorize the users’ locations. Figure 7 shows the distribution of all annotated locations during our user study among all users.

**Figure 8:**
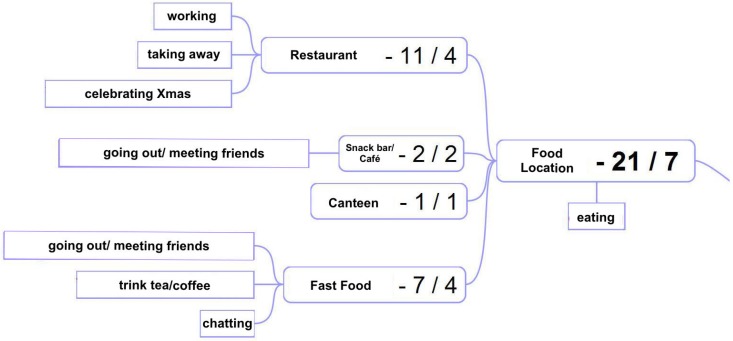
Multiple purposes of visiting a food location (beyond eating).

What is interesting about the data is that often the same location type comes with more than one purposes of visiting that location. That is, users often entered different high-level activities for the same location based on the respective context each time. Figure 8 illustrates this in the case of the location type *food*.

**Figure 9:**
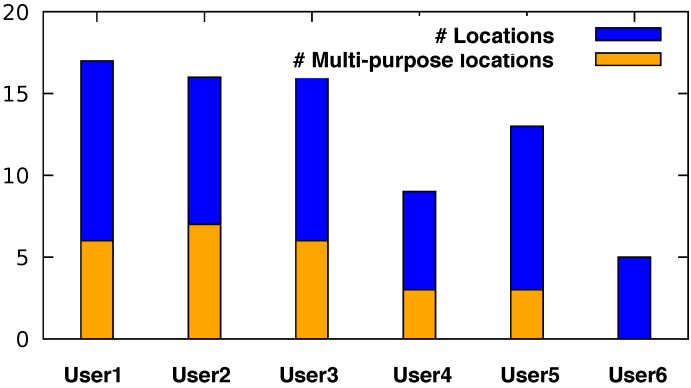
Number of (unique) multipurpose locations proportional to the total number of (unique) locations per user.

We can see that the users entered 21 different reasons for visiting a food location (including the respective sublocations). Apart from the obvious purpose “eat and drink”, some participants visited a restaurant just shortly to get some take away food, some were working there and some visited a restaurant for socializing reasons to meet with friends and/or celebrate Christmas with them. It is obvious that each of the aforementioned purposes of visiting a certain place influences the visiting duration and attendance times in general, which in turn affects the overall movement patterns of the users. This underpins our primary idea of using the users’ high-level activity to extend the spatio-temporal Markov model. A person working in a restaurant shows a regular visiting pattern in specific days and times, while a person who is just passing by to get some take away food will stay there just for a few minutes (ideally). Furthermore, based on common sense knowledge, one could conclude that the latter person would probably take the food and go directly home instead of visiting a club. Figure 9 shows the proportion between the total number of the various (unique) semantic locations per user to the number of the locations at which more than one purposes of visiting that locations was entered.

**Figure 10:**
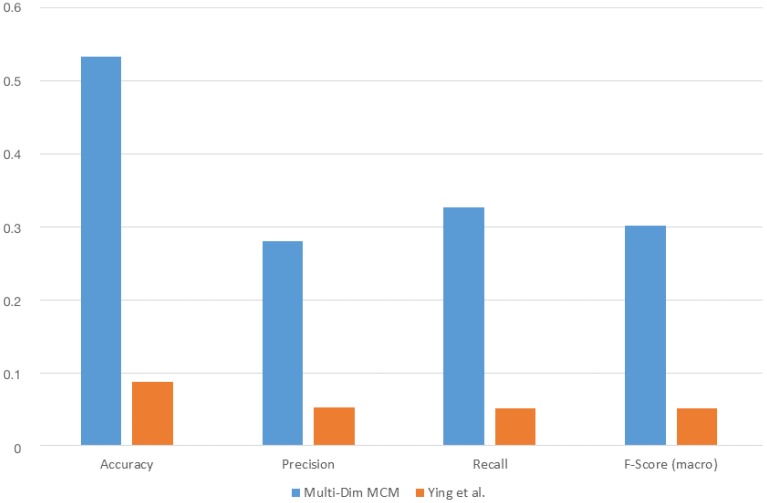
The 1. order multi-dimensional Markov Chain model vs. Ying et al.’s approach with regard to accuracy, precision, recall and F-score.

We chose the k-fold cross validation to be our evaluation method and tested the following k-parameter values: [5, 10, 15, 20]. The results in this section refer to a k-value of 20. In addition, we compared our multi-dimensional 1. order Markov Chain model with the semantic trajectory based approach of Ying et al. [6] with a minimum support of 0.01. Figure 10 shows how the multi-dimensional Markov Chain model performs against Ying et al.’s with regard to accuracy, precision, recall and F-score.

We can see that the Markov model outperforms Ying et al.’s framework in all four cases. The absolute values are however generally low. This can be mainly attributed to the small size of our training dataset. The collected data are not able to cover all the situations contained in each Markov Chain, which leads to a reduced performance. A solution for this issue is discussed in Section 6.

The approach of Ying et al. seems particularly sensitive to the size of the available data. This can be explained by the fact that their core algorithm is based on mining semantic trajectories out from the data based on their frequency of occurrence. In the case of a small dataset, there are fewer trajectories to be found. Therefore, the necessary pre-fix trees on which their approach relies cannot be built. This results in a constrained set of possible future locations from which their predictor can chose from and thus to a reduced performance.

## 6. Purpose-of-Visit-Driven Semantic Similarity (PoVDSSA) on Semantic Trajectories

Thus far, we explored whether and to what extent the semantic level used for describing the locations affects the modelling and predictive performance of a 1. order Markov model. It could be shown that trajectories of higher semantic level can lead to more accurate predictions. On the one hand, this can be attributed to the fact that people’s regular movements rely mainly on high-level patterns and rules. On the other hand, high-level trajectories contain fewer classes for the Markov model to predict as the lower-level ones, a fact that supports additionally the prediction performance. Then, we extended the spatial model and built a multi-dimensional context-aware Markov Chain construct for modelling semantically enriched trajectories. Our approach takes, beside the current (semantic) location, time of day, day of week and the users’ current activity into consideration. We evaluated our idea on a real-word dataset and compared it with a state of the art semantic trajectory based algorithm. The multiple Markov Chain construct outperforms the baseline by yielding a higher performance in terms of accuracy, precision, recall and F-score. However, a certain sensitivity towards the dataset size could also be identified. In this last part of this work, we propose a solution against this limitation.

**Figure 11:**
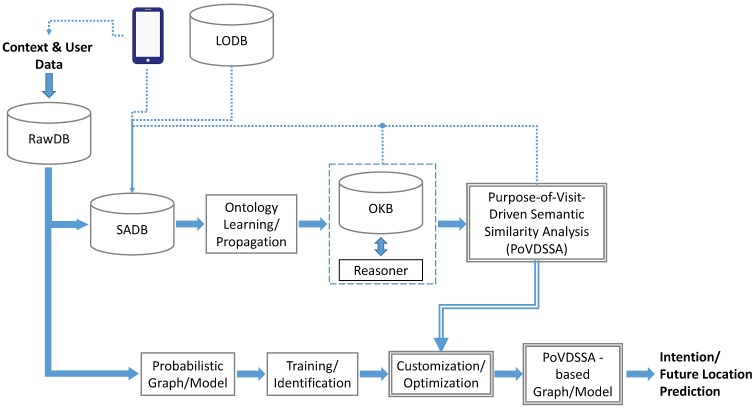
Purpose-of-Visit-Driven Semantic Similarity Analysis based location prediction (PoVDSSA) framework, where rawDB refers to the raw data Database, SADB refers to the Semantically Annotated Database, LODB to a Linked Open Database, OKB to the Ontology-based Knowledge Base and PoVDSSA to the Purpose-of-Visit-Driven Semantic Similarity Analysis Component, respectively.

The framework we propose is based on a similar architecture that was first introduced in [4] and is illustrated in Figure 11. Our approach merges semantics with machine learning and consists of two main parts. One part is responsible for the semantic processing of the available information (top branch), while the other one takes charge of the actual location prediction (bottom branch).

The (sensed) datas such as location and time (e.g., GPS readings) follow both paths at the same time. On the one hand, these are being semantically annotated and together with further semantic information, such as the purpose of visit, stored in the Semantically Annotated Database (SADB). The annotation of locations takes place semi-supervised partly by the user (through an Android app running on the smartphone (see Section 5.1.1), and partly by utilizing a (geographic) Linked Open Database (LODB). In this way, both public as well as private locations, such as the users’ home or work, can be correctly identified among the recorded data. The same tracking Android app is also responsible for collecting additional semantic information such as the purpose of visit mentioned above. The resulting data are then used to propagate our Ontology-based Knowledge Base (OKB) described in Section 6.1 and to build our so-called *Purpose-of-Visit-Dependent Frame* objects (*PoVDF*). A reasoner assists additionally the creation and the extension of our ontology in the case of lacking data by making use of the existing assertions (e.g., by means of subsumption reasoning based on Description Logic (DL)). *PoVDSSA* is the core component of our approach and refers to the *Purpose-of-Visit-Driven Semantic Similarity Analysis* that takes place in order to cluster locations dynamically depending on the respective purpose of visit. Thus, it is responsible for providing our approach with a dynamic context-aware view of locations at anytime. A detailed description of PoVDSSA can be found in Section 6.2.

**Figure 12:**
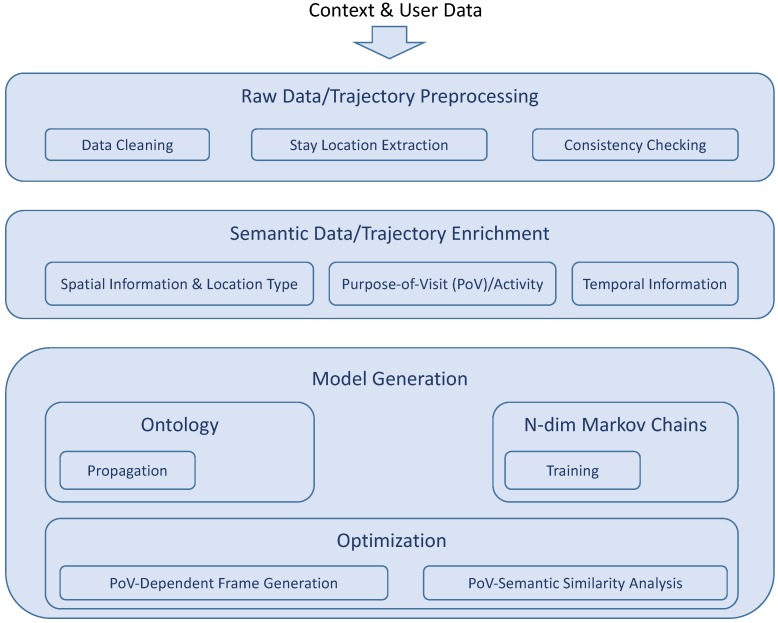
Layer diagram of the Purpose-of-Visit-Driven Semantic Similarity Analysis based location prediction framework (PoVDSSA).

The bottom branch of our framework contains the actual location prediction model, which is in our case the multi-dimensional Markov Chain ensemble described in Section 5. In other words, the presented method here represents an extension of the former model. Other machine learning techniques such as Artificial Neural Networks (ANNs) can be applied here as well though, as shown in [41]. The training and prediction process is as follows. First, the prediction model is trained with the available raw data clustered into significant locations, such as in [7]. Next, the trained model expects an optimization through the customization of its (previously learned) parameters based on the semantic similarity analysis of locations described in detail in Section 6.3. Finally, the optimized PoVDSSA-based prediction model is able to provide an estimation about the future locations that the user(s) intent to visit next. Figure 12 summarizes the principal functions of our approach into a set of three layers: the preprocessing layer, the semantic trajectory enrichment layer and the modelling and optimization layer.

### 6.1. Semantic Enriched Location Data and Ontology-Based Knowledge Base

To comprise all the different facets of the associated to the locations semantic knowledge, we chose to use an ontology-based knowledge base. In this way, we are able to represent hierarchical and taxonomical relations of locations, purposes of visit, high level activities and elementary actions, as well as to define our own properties and relations to one another. At the same time, we use the same ontology to describe temporal information as well. We implemented our ontology in OWL using the Protege tool (http://protege.stanford.edu). Our ontology consists of four major entities (classes):

1. Locations
2. Purpose of Visit
3. Actions
4. Temporal and Event

The *Locations* entity captures a taxonomy of various location types, like *night club, bar, restaurant, dinner, fast food restaurant*, etc. To build the particular taxonomy, we oriented ourselves on the Foursquare venue categorization (https://developer.foursquare.com/categorytree).

The *Purpose of Visit* entity covers the annotated reasons for visiting each location. In tangible terms, it refers to the complex, high-level activities that may take place in a location, such as *working*, *celebrating* and *relaxing*.

The *Actions* entity includes the elementary actions of which the high-level activities are composed. For instance, the high-level activity *celebrate a birthday* is related to the low-level actions *meet friends, meet family, eat, drink, etc*.

**Figure 13:**
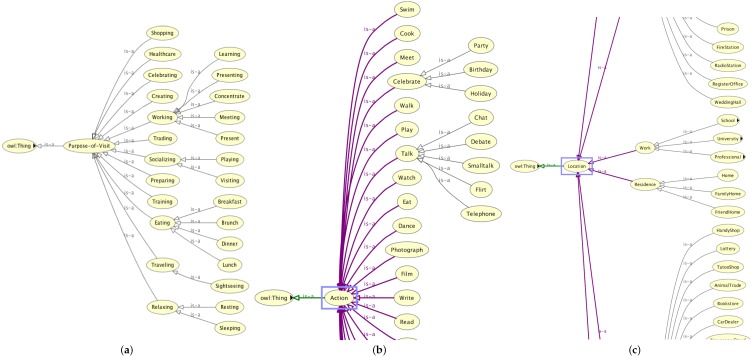
(**a**) Purpose of Visit entity; (**b**) part of the Actions entity; and (**c**) part of the Location entity.

Finally, the *Temporal and Event* entity describes time from a human point of view, considering a human-like time granularity. For describing duration (time intervals with start and end) and time in general, we made use of the standard OWL Time Ontology (https://www.w3.org/TR/owl-time/#toc). This is necessary, inter alia, for defining timeslots and blocks which in turn provide the temporal granularity of our semantic trajectories. Beside time in general, particular attention is paid to the temporal entity event, which refers to special events such as *anniversaries, birthdays, public holidays*, etc. that are strongly related to irregular behaviour. Figure 13a–c illustrates some parts of the aforementioned classes in our ontology.

**Figure 14:**
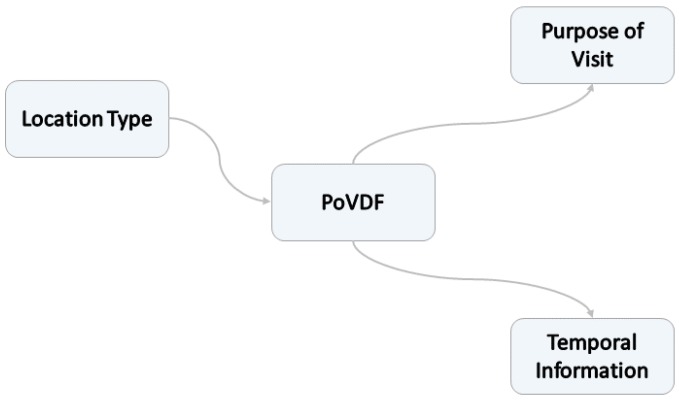
Introducing a new entity PoVDF for representing an n-ary relation.

As already mentioned in Section 1, the location prediction framework introduced in this section aims at capturing the dynamic role of locations based on the respective current context. For this purpose, we need to go beyond using just the location type and link the location entity with the rest of the concepts of our ontology: the time, the high-level purpose of visit and the corresponding activities. Moreover, we consider this context information as additional attributes of the respective location. However, OWL supports solely *binary relations*, that is, relations between two individuals. Relations that handle more than two individuals are referred to as *n-ary relations*, with n>2. To link more than two individuals, there exists a number of workaround solutions, so called *Ontology Design Patterns (ODP)* [42]. One way to overcome the binary relation problem is to introduce a new class for representing the desired relation [43]. In this work, we define a new entity, which we name *Purpose-of-Visit-dependent Frame (PoVDF)* (see Figure 14).

The underlying idea is to use this class for clustering locations that belong together due to sharing common attributes, such as the same reason for visiting them. The name refers in part to Minsky’s cognitive *Frames* from the 1970s, which uses this term to encapsulate situation- and experience-based knowledge [44,45].

### 6.2. Purpose-of-Visit-Driven Semantic Similarity Analysis (PoVDSSA)

Humans tend to employ cognitive frames, that is certain mental constructs, to interpret things, entities and their experiences about them [46]. Taxonomization and building groups and relations between them and the included entities help clarifying concepts and are therefore of high importance to us. To build groups, i.e., to *cluster* things, we draw on the fundamental notion of *Similarity*. Similarity is defined as follows [47]:

Having a resemblance in appearance, character (characteristics), or quantity, without being identical.

According to this definition, two objects are similar, when they share the same characteristics. While this definition refers rather to the similarity between two physical objects, it can analogously be extended to a more general one that expresses a characteristic-based similarity between two objects in a knowledge graph or an ontology. This kind of similarity can then consequently be referred to as *semantic similarity*. Likavec et al., inspired by Tversky’s work [28], defined and investigated such a property-based semantic similarity among ontological objects [48].

In our work, we adopt Likavec’s method and define a similar equation to cluster semantically the visited locations of the users based on the purpose of visit and the corresponding time. Thus, we treat both the time, and the purpose of being at a location as characteristic features of that particular location, which in turn reflects the PoVDF concept mentioned in Section 6.1. Equation (4) illustrates the property-based semantic similarity adapted to our use case:

(4) $$Sim\left(l_{1},l_{2}\right)=\frac{CP(l_{1},l_{2})}{DP\left(l_{1}\right)+DP\left(l_{2}\right)+CP\left(l_{1},l_{2}\right)},$$

where $l_{1}$ and $l_{2}\in L$ represent two different locations, CP refers to the common properties (e.g., purpose of visit, time and day) of the particular locations and DP gives the distinctive purposes that are associated only to the one location and do not appear in conjunction with the other. However, Equation (5) captures solely a single moment. That is, it actually describes the similarity between two particular *stays* $s_{1}$ and $s_{2}$ at locations $l_{1}$ and $l_{2}$, respectively, only for a certain moment and not the overall location similarity. A *stay* refers here to a single visit of a certain location at a certain time (and day) for a certain reason. To calculate the overall semantic similarity between locations, we compute the average pairwise similarity of all existing stays at $l_{1}$ and $l_{2}$, as shown in Equation (5). This reflects our definition of a *Purpose-of-Visit-Driven Semantic Similarity (PoVDSS)*.

(5) $$PoVDSS{\left(l_{1},l_{2}\right)}_{pair\emptyset }=\frac{\sum_{i=1}^{N}\sum_{j=1}^{M}Sim\left(s_{i},s_{j}\right)}{M*N},$$

where *M* and *N* provide the number of stays at location $l_{1}$ and $l_{2}$, respectively. Our PoV-driven semantic similarity takes all different location and purpose-of-visit hierarchy levels that are modelled in our ontology into consideration and calculates a max-min normalized aggregated value between 0 and 1.

By clustering locations in this way, we are able to go beyond a simple type-specific categorization of locations and cluster even locations of different type together. Let us consider an example to clarify this statement. The locations *park*, *gym* and *restaurant* would probably land in three different categories if we tried to cluster them by taking only the location type into account. In contrast, our approach provides a more dynamic clustering by also considering the purpose of visit. In this case, *park* and *gym* would be considered temporarily similar if the person visits the park for jogging as jogging is a fitness activity and thus common to the gym’s overall purpose of visit. Analogously, *park* would be found similar to *restaurant* if the person has a picnic at that park.

### 6.3. PoVDSSA-Based Model Optimization Process

This section describes the optimization process for the multi-dimensional Markov Chain model of Section 5, which we use here as our location predictor. In this case, the optimization process adapts and updates the location transition probability matrix based on the Purpose-of-Visit-Dependent Semantic Similarity Analysis (PoVDSSA) illustrated in the previous section.

The PoVD semantic similarity analysis takes place each time when a prediction is to be made. It investigates how similar the current location $l_{cur}$ is to each of the other locations, that is, location types found in our propagated knowledge base. At the end, it provides us with a number of locations and their corresponding similarity scores. Next, we rank the results based on their score. The top location represents the current location itself because it corresponds to an absolute similarity of 1.0 and thus we disregard it and choose the second from the top location as the most similar location $l_{maxSim}$. At each prediction time step, we weight the Markov matrix’ transition probability row of the location $l_{maxSim}$ with the highest similarity to the current location $l_{cur}$ by multiplying each element with the maximum similarity score ($SimScore_{max}$). Finally, we use the resulted row to update the transition probability row that corresponds to the current location by applying the following formula:

(6) $$\begin{array}{cc} TP{\left(l_{cur}\right)}_{i,new}\;=\; & TP(l_{maxSim})\times SimScore\\ & +offset\times TP{\left(l_{cur}\right)}_{i,old} \end{array}$$

However, the transition probability is updated only if the respective similarity score exceeds a certain threshold $min_{Sim}$. The updating algorithm is described in detail below.

Thus, the updated transition probabilities for the current location depend, on the one hand, on the transition probabilities of the most similar location and the corresponding similarity score. On the other hand, they still depend on the old values, although much less now due to the *offset* factor. This provides us with a smooth and adaptable reward-penalize function.

## 7. Evaluation

To evaluate our approach, we used the preprocessed data collected during the user study described in Section 5.1.1. We used k-fold cross validation for our evaluation and tested the following k-parameter values: [5, 10, 15, 20]. We compared our approach with a multi-dimensional semantic 1. order Markov Chain Model as well as with the semantic trajectory-based approach of Ying et al. [6] with a minimum support of 0.01 that we use here as reference.

**Figure 15:**
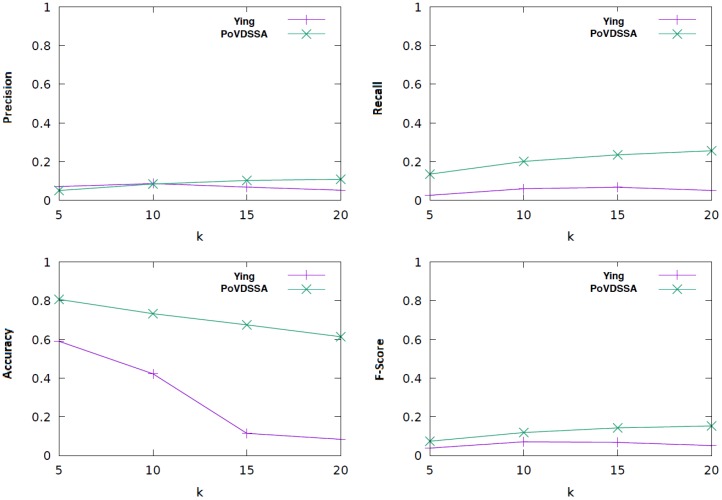
Comparison of our approach (PoVSSA) to Ying et al.’s approach with regard to accuracy, precision, recall and f-score.

Figure 15 illustrates the performance of our PoVDSSA-based approach in comparison to Ying et al.’s framework over various *k* values with respect to (macro) accuracy, (macro) precision, (macro) recall and (macro) F-score.

As can be seen in the figure, our approach performs better than Ying et al.’s framework almost every time in terms of all four metrics. It leads clearly to both a higher accuracy performance and a higher recall value. This means that our system is not only more accurate, but also its estimations are more consistent, especially when the *k* value is high. That is, it seems that it is easier for our model to find the relevant locations among the available locations in the test dataset during the estimation process. This can be mainly attributed to the fact that our approach is able to replace and fill out missing current location transitions through existing transitions coming from the corresponding similar location. In this way, it can handle the data sparsity problem mentioned in Section 5. The accuracy in both methods is decreasing if we use a finer data breakdown (higher *k* value). This can be explained by the fact that our data consist of sequences. The finer we split the sequences, the more incoherent (in terms of chronological order) become the resulting training data. Thus, it becomes harder for the models to train. Furthermore, the upper left of the figure shows that our approach is only slightly better than our baseline with regard to precision. This reflects the fact that both share the same ratio of predicting the respective future locations correct and incorrect (True positives and False positives, respectively). In other words, the estimations of both models scatter at a comparable level. Apart from the accuracy, the models show overall low performance scores. These can be mainly attributed to the size and the quality of our training and evaluation dataset.

**Figure 16:**
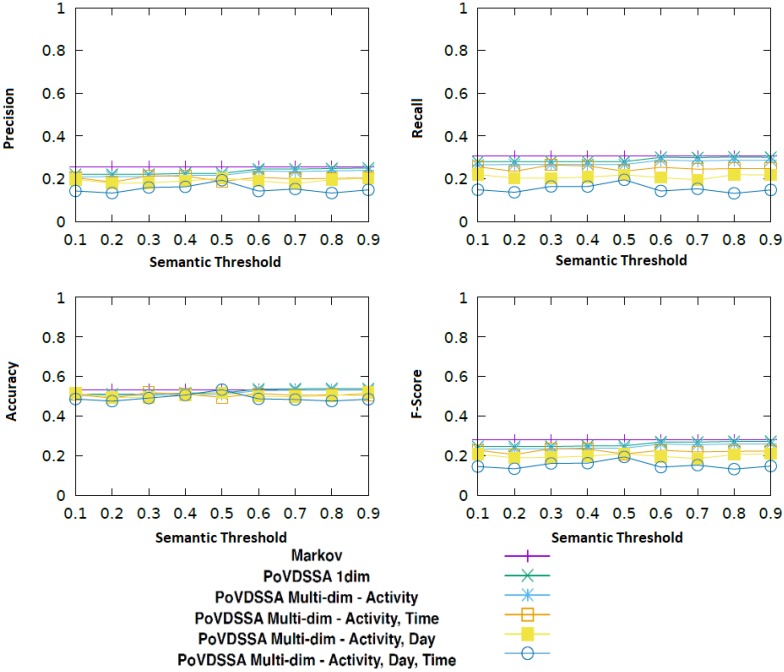
Comparison of various different configurations of our approach (PoVSSA) to the Markov model described in Section 5.

Figure 16 displays the performance of different configurations of our approach compared to the Markov model described in Section 5 in relation to the similarity threshold value, which determines whether an updating should occur (see Algorithm 1).

**Algorithm 1:** Markov transition probability updating process.

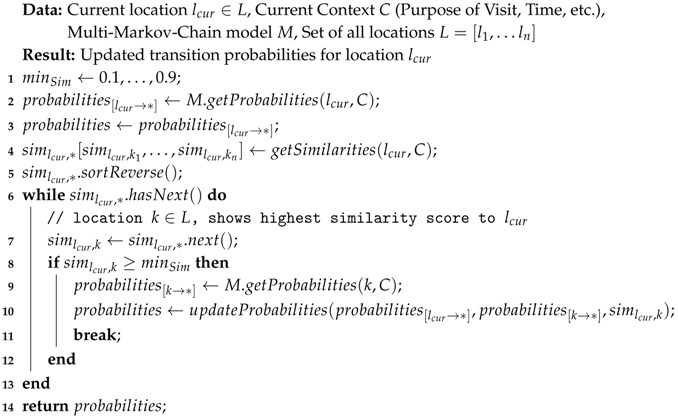



In particular, we use the Markov model as baseline and we explore the impact of the diverse dimensions on the recall performance of our PoVDSSA-based approach.

- *PoVDSSA 1-dim*: a model that considers solely the location
- *PoVDSSA Mutli-dim-Activity*: a model that considers location and activity
- *PoVDSSA Multi-dim-Activity, Time*: a model that considers location, activity and time of day
- *PoVDSSA Multi-dim-Activity, Day*: a model that considers location, activity and day of week
- *PoVDSSA Multi-dim-Activity, Time, Day*: a model that considers location, activity, time and day

We can see that most variants of our model achieve a similar performance as our baseline, without being able to outperform it. In terms of only accuracy, all models perform equally, with the one-dimensional PoVDSSA-based model achieving a slightly higher score. Interestingly, with respect to recall, precision and consequently F-Score, increasing the number of dimensions seems to have a negative effect, i.e., the higher is the dimension number, the worst is the performance. Especially the additional temporal dimensions (time of day and day of week) seem to lead to lower scores. The spatial (PoVDSSA 1-dim) and the Location–Activity (PoVDSSA Multi-dim-Activity) model yield the overall best results compared to the rest of the model variants. At the same time, the model that takes all the available context information (location, activity, time of day and day of week) into account shows the poorest performance.

What also stands out in Figure 16 is that the value of the semantic similarity threshold also seems to be playing a significant role, particularly for the non-temporal models, the models that do not take temporal information into account (PoVDSSA 1-dim and PoVDSSA Multi-dim-Activity). In the case of the latter, a higher threshold leads to better results. In tangible terms, this means that models that lay more weight in the similarity between locations achieve better results, when these models do not take temporal information into account. However, a too high threshold limits the number of the existing potential similar transitions, which in turn downgrades the performance.

Using time and day as class attributes for comparing two location types appears to be not useful. On the contrary, it seems to have a negative influence. In particular, the information *day of week* appears to be more critical than the information *time of day*. This can be attributed to the size of our dataset. It contains movement patterns of five weeks. This means that there exist only five daily trajectories for each single day of the week. This number is too low for our model to be able to make serious assumptions based on the day of week. In comparison, each hourly slot exists 35 times in our dataset.

In general, at first glance, the updating of the location transition probability matrix based on the semantic similarity of the respective locations does not seem to be leading to any kind of improvement. Moreover, it seems that our models face largely the same difficulties as our non-semantic-enhanced Markov model, the sparsity of our collected dataset. After further analysis of our results, we found that the uneven distribution of the labelled data among users, the overall missing or false labelling of the purpose of visit and the fact that our five-week long data cover very few location–time–day–activity combinations have led to a situation in which our PoVDSSA-based updating approach was triggered only very rarely—too rarely to cause some significant positive impact.

**Figure 17:**
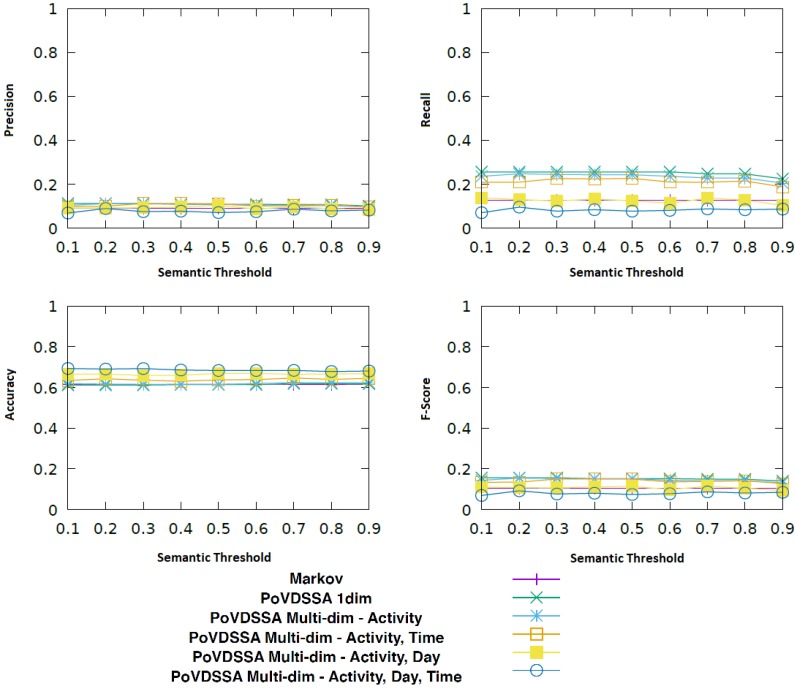
Comparison of various different configurations of our approach (PoVSSA) to the Markov model described in Section 3.2 for the user with the most annotated locations and activities.

However, our analysis also showed that the larger and more consistently annotated the dataset is, the better our approach performs. Figure 17 refers to the results of the user with the most annotated locations and activities.

In this case, almost all of our PoVDSSA-based variants perform better than our baseline. Once again, the *PoVDSSA 1-dim* and the *PoVDSSA Multi-dim-Activity* outperform the rest, achieving an almost twice as high recall and F-Score value as the (multi-dimensional) Markov approach in Section 5. Furthermore, as before, the model that considers the most context information (*PoVDSSA Multi-dim-Activity, Time, Day*) shows the worst performance and lies with respect to precision, recall and F-Score below the Markov baseline. However, surprisingly, this time, in terms of accuracy, it provides, with almost 70%, the highest results, a fact that at least indicates the importance and the added value in taking additional context information into account. It is also apparent in the figure that in this case the semantic similarity threshold plays a less significant role.

Overall, we could see that our Purpose-of-Visit-Dependent approach is capable of leading to overall better results than the Markov in most cases with a consistently labelled dataset. However, it shows a similar sensitivity towards training data sparsity and quality as the multi-dimensional Markov model of Section 5. We believe though that with a bigger dataset the performance gap would tend to favour the PoVDF-based approach.We could also see that temporal information actually impairs our system instead of improving it. This can be explained by the fact that, when it comes to human movement, it is the sequence itself which plays the most significant role and not the absolute information of time or day. This could be further explained by our sparse dataset, which does not cover all *time–location–activity* combinations. We would expect better results with more data that cover much more different situations in the users’ real life.

## 8. Conclusions and Future Work

In this work, we evaluate the performance of three Markov Chain based models in modelling semantic trajectories and predicting future locations. First, we investigate the role of the semantic representation level of locations in the predictive performance of a spatial Markov model. We could see that the semantic level indeed plays a significant role with high-level models performing better than the lower-level ones. Then, in the second part of our work, we test an extended Markov model that incorporates additional context information in terms of time, day and the user’s activity. It can be shown that, to a certain degree, this additional knowledge can help the probabilistic model to achieve higher scores. However, we could also identify some limitations, attributed mainly to the sparseness of our training and evaluation dataset. To solve this issue, in the last part of our work, we propose to add a *Purpose-of-Visit-Driven Semantic Similarity (PoVDSSA)* component to our multi-dimensional Markov Chain model. We were driven by our hypothesis that dynamic situation-dependent location clustering can enhance the overall performance. We implemented our PoVDSSA-based algorithm and tested it on a real life dataset, which we collected ourselves by conducting a mobile user study. We evaluated our framework in contrast to two other approaches, namely to Ying et al.’s semantic trajectory based approach and a conventional Markov Chain model. We show that our approach outperforms both Ying et al.’s approach and the Markov-based approach. However, some drawbacks, due mostly to the lack of a bigger dataset, could also be identified. In the future, we plan to implement an extended and more personalized version of the presented context-aware semantic similarity analysis approach that takes personality and emotions additionally into account to optimize the predictive performance of our model.

## Figures and Tables

**Table 1:** Ten-fold cross validation average and maximum accuracy results of the 1. order Markov model for the 12-week single-user dataset for both the top ($Level^{\left(1\right)}$) and the lower semantic level ($Level^{\left(3\right)}$).

	Avg Accuracy	Max Accuracy
*High Level* ^(1)^	0.750	1.00
*Low Level* ^(3)^	0.611	0.942

**Table 2:** Ten-fold cross validation average accuracy, maximum accuracy and weighted F-Score results of the 1. order Markov model for the eight-week multi-user dataset for both the top ($Level^{\left(1\right)}$) and the lower semantic level ($Level^{\left(3\right)}$).

	Avg Accuracy	Max Accuracy	F-Score (Weighted)
*High Level* ^(1)^	0.385	0.559	0.386
*Low Level* ^(3)^	0.301	0.448	0.302
